# ‘Simply do it.’: Results from an online questionnaire to inform a community-based menopause education and support programme in the UK, InTune

**DOI:** 10.1177/20533691251372818

**Published:** 2025-09-09

**Authors:** Joyce Catherine Harper, Nicky Keay, Mehab Mir, Annice Mukherjee, Jane Plumb, Geeta Kumar, Janet Lindsay, Jeremy Barratt, Sophie Strachan, Shema Tariq

**Affiliations:** 1 Institute for Women’s Health, 4919 University College London, London, UK; 2 Division of Medicine, 4919 University College London, London, UK; 3 40368 Coventry University, Coventry, UK; 4 4845 Royal College of Obstetricians and Gynaecologists, London, UK; 5 59405 Wellbeing of Women, London, UK; 6 Sophia Forum, London, UK; 7 Department for Infection and Population Health, Insititute for Global Health, University College London, London, UK

**Keywords:** Menopause, perimenopause, postmenopause, education

## Abstract

**Objective:**

We are developing a menopause education and support programme, ‘InTune’, using co-design that includes focus groups and workshops. We have identified the need for two key interventions: one aimed at raising general awareness, *Be Prepared for Menopause*, and another to support those currently experiencing menopause symptoms. This survey aims to reach a broader audience to better understand their needs and preferences around menopause education and support. The insights gained will help us further develop the InTune programme.

**Study design:**

An anonymous, online cross-sectional questionnaire was developed with key stakeholders, covering demographic characteristics, menopause preparedness, and opinions and recommendations for programme design. The survey was conducted online using Qualtrics between 16/1/24 and 22/3/24.

**Main outcome measures:**

Of 1596 respondents (98.4% female; 75.6% White British; median age 50 years; 79.5% peri/postmenopausal), 77.4% reported being somewhat informed or not informed about menopause. Over 90% agreed that a national menopause education and support programme was needed, delivered in-person and/or online, in weekly hour-long sessions, over 2–4 weeks. Key to acceptability and success is that information is accessible, accurate, and based on ‘latest scientific evidence and debunk[s] some of the myths’. Respondents emphasised the importance of developing a programme that was accessible to all, regardless of gender, ethnicity, age, sexuality, disability status, neurodivergence and age at/cause of menopause.

**Conclusion:**

Over 90% of participants agreed that a menopause education and support programme with standardised and evidence-based content was needed. This data will allow us to refine the InTune programme.

## Introduction

Menopause is a hormonal and social shift that all women (and anyone with ovaries) experience at some point. There are an estimated 13 million people in peri- and postmenopause in the UK at any one time, equivalent to one third of the population.^1^ This transition, usually a result of age-related decline in ovarian function (but also occurring because of surgery or medication), lasts several years and can result in a variety of symptoms. The most commonly reported symptoms are vasomotor, with a prevalence of up to 80%.^2^ Other symptoms include sleep disruption, mood and cognitive changes, loss of libido and urogenital symptoms such as vaginal dryness and urinary incontinence.^3^

Awareness and understanding of menopause, and its associated symptoms and potential impacts, can help individuals prepare for this important transition. A review of psychosocial interventions in peri- and postmenopausal women found that interventions comprising psychoeducation, health education and/or health promotion reduced negative attitudes towards menopause, reduced menopause-associated symptoms, increased healthy lifestyle behaviours, and improved overall physical and psychological wellbeing.^4^ Yet despite this, there remains a paucity of evidence-based, theoretically informed and rigorously evaluated public health programmes targeting individuals in peri- and postmenopause.

The neglect of menopause by public health systems in the UK has resulted in a large number of people reaching this stage of life feeling unprepared. In 2021, the Department for Health and Social Care (DHSC) in England launched a public consultation for their national Women’s Health Strategy, finding that the overwhelming majority of respondents (91%) felt they lacked sufficient information about menopause.^5^ A survey of 3143 women by the lead author (JH) found that over 50% of peri-^6^ and postmenopausal^7^ women felt they had not been informed about menopause at all, with the majority not thinking about menopause until they reached their 40s. Nearly a third of perimenopausal respondents reported ‘dreading’ the menopause.^8^ The majority of women in this study (68%) reported looking for information only after their symptoms had started, with friends, general practitioners (GPs) and websites being the most common cited places to seek information. Similar results were obtained from women under 40.^9^

There has been a rapid expansion in unregulated private companies and individuals providing menopause information and support for profit^10^; this has been termed the ‘menopause gold rush’.^11^ This fragmented landscape of menopause support and education leaves people vulnerable to financial exploitation, may propagate misinformation and is likely to amplify existing menopause-related health inequities.^5,12–14^

There is a recognised need for high quality, accessible, evidence-based and standardised menopause health education and health promotion programmes that take into account the diversity of menopause experiences.^10,15^ Our multidisciplinary team (comprising academics and clinicians) seeks to develop InTune (United Kingdom Menopause Education and Support for Everyone), the UK’s first ever national menopause education and support programme.^16^ From our work so far with focus groups and workshops, we have identified two education and support interventions that are needed which we have started to co-design with diverse communities affected by menopause. Both interventions will be composed of videos by UK leaders in menopause who will explain the key topics of menopause combined with interactive group discussions led by trained facilitators. The first we have called Be Prepared for Menopause, and we are trialling a 2 h event aimed at women under age 40 to help them identify when they reach the perimenopause, but this event is suitable for everyone, including men. The second intervention, which is still in development, is aimed at people experiencing menopause-related symptoms. Drawing upon models of antenatal classes, this will combine more detailed videos on key topics related to menopause and peer support, whereby people attending sessions, face-to-face or online, will share experiences, information and strategies to foster understanding, empowerment and community.^17^

As part of our co-design process, in this study we have consulted members of the public on menopause education and support needs, curriculum content and preferred programme delivery, using an online questionnaire to reach a wider audience than our focus groups and workshops. We aimed to investigate current menopause education and support needs and preferences, among individuals who have experienced or who could experience menopause transition.

## Methods

### Study design

This was primarily a quantitative cross-sectional questionnaire with some free-text responses. It was administered online using Qualtrics (Qualtrics, Provo, UT) between 16th January and 22nd March 2024.

The questionnaire was designed by the authors (JH, NK, ST) and informed by the team’s previous research, existing literature, project workshops and focus group discussions. It was further refined through discussions with our Expert Advisory Group.^16^ It covered demographic characteristics, menopause preparedness and opinions and recommendations for programme design (supplemental file), with space for free-text responses. We tested the questionnaire with 40 volunteers in January 2024. As no major changes to questionnaire design were required, we included these responses in this paper.

### Recruitment

We recruited participants online purposively via professional and personal networks, and social media of the authors (WhatsApp, Facebook, Instagram, X/Twitter and LinkedIn). To take part, people had to give electronic informed consent (after reading an information sheet) and confirm they met inclusion criteria (age ≥18 years and currently experiencing or could potentially experience menopause (including being a woman, transgender man, or non-binary person)). The survey was only available in English but was open to non-UK residents.

### Data analysis

Only those respondents who consented and submitted their questionnaires were included which allowed people to withdraw before submission. Quantitative and qualitative data were kept analytically distinct. We used qualitative data to contextualise quantitative findings, and for triangulation with quantitative findings. Quantitative data were analysed in Stata (StataCorp. 2023. *Stata Statistical Software: Release 18*. College Station, TX: StataCorp LLC) to produce descriptive statistics and bivariate analyses. Free-text responses were imported into the qualitative data analysis software NVivo and analysed thematically using a reflexive thematic approach.^18^ This involved familiarising ourselves with free-text responses, generating initial codes, and then categorising into sub-themes and themes. Any disagreements about coding and/or interpretation were discussed until consensus was reached. Findings were discussed among the team, and feedback elicited from Expert Advisory Group members.

### Positionality statement

We acknowledge that values, and assumptions as researchers, which are shaped by our social identities and life experiences, will influence our research questions, methodological approaches, and interpretation. The work reported in this paper was primarily designed, conducted and analysed by JH (a cisgender, mixed-race, postmenopausal woman with a professional background as an academic in reproductive health, and ST (a cisgender, British Pakistani, perimenopausal woman with a professional background as a sexual health physician and public health academic). Both JH and ST have led research on menopause for 10 years. Preliminary analysis of data was undertaken by MM (a cisgender, Pakistani, premenopausal woman and MSc student).

### Ethics

Ethical approval for this study was granted by the UCL Research Ethics Committee (reference 9831/012).

## Results

Between 16th January and 22nd March 2024, 2002 people consented but only 1596 responses that were submitted have been included for analysis. Almost all participants described themselves as female (*n* = 1562, 98.4%); 75.6% were of White UK ethnicity (*n* = 1195), and the majority lived in the UK (*n* = 1507, 94.5%) (Table 1). The median age of participants was 50 years (interquartile range (IQR) 45–55 years); the majority reported being peri- (*n* = 687, 43.2%) or postmenopausal (*n* = 577, 36.3%). Over a quarter (*n* = 425, 26.6%) reported living with a disability and/or long-term condition. The majority (*n* = 1250, 79.1%) were university-educated, and over 95% (*n* = 1510, 95.6%) reported having enough money to meet their basic needs all or most of the time.

**Table 1.** Participant characteristics.

	n^a^(%)
Age in years (*n* = 1597) median, IQR	50 (45.55)
Gender	
Female	1562 (98.4)
Non-binary	21 (1.3)
Transgender man	1 (0.1)
Other	3 (0.2)
Ethnicity (n = 1581)	
White British	1195 (75.6)
Other White	197 (12.5)
Asian/Asian British	81 (5.1)
Black/Black British/Black African/Black Caribbean	29 (1.8)
Mixed	47 (3.0)
Other	32 (2.0)
Sexuality (*n* = 1590)	
Heterosexual	1399 (88.0)
Lesbian/gay	41 (2.6)
Bisexual	83 (5.2)
Other (including pansexual and asexual)	34 (2.1)
Prefer not to say	33 (2.1)
Disability status (n = 1596)	
No disability	1171 (73.4)
Physical or mobility impairment	68 (4.3)
Long-term condition	235 (14.7)
Sensory impairment	37 (2.3)
Learning difficulty/cognitive impairment	64 (4.0)
Autism	44 (2.8)
Highest level of education (*n* = 1581)	
None/primary	3 (0.2)
Secondary/college	271 (17.1)
University (including postgraduate)	1250 (79.1)
Other	57 (3.6)
Basic needs met (n = 1579)	
All/most of the time	1510 (95.6)
Some/none of the time	69 (4.4)
Menopausal Status^b^ (*n* = 1589)	
Premenopausal	149 (9.4)
Perimenopausal	687 (43.2)
Postmenopausal	577 (36.3)
Not sure	176 (0.1)

^a^Numbers vary due to non-response.

^b^self-reported.

### Menopause informational and support needs

Nearly two-thirds of participants (*n* = 992, 63.1%) reported negative feelings about peri- and/or postmenopause; only 5.3% (*n* = 83) reported having positive feelings towards this stage of life. Nearly one-quarter (*n* = 346, 21.9%) reported feeling well informed about the menopause transition, with 56.6% (*n* = 896) and 20.8% (*n* = 329) stating they were either somewhat informed or not at all informed, respectively. Several participants shared the challenges they faced in actively searching for information that was relevant to them. The broad range, and often non-specific, nature of menopause-related symptoms added to the difficulty of navigating this process.

‘[I] have to seek out information relevant to you from different sources. So many symptoms linked to menopause never know what is attributable to menopause and what is not. Limited/untested options about what to do about them’.

Some highlighted the significant effort required to find reliable information about menopause and distinguish facts from misinformation:

‘I have found info very useful but I have done lots of research myself, so I am quite discerning in what I read/believe. There is a lot of nonsense/incorrect info floating around in the media too’.

Participants described existing information as variable in quality, leading to mistrust and confusion:

‘The info out there is of varying quality, some is rubbish, some is backed by science, some is intended just for marketing to sell supplements etc. It must be difficult for many women to know what to rely on’.

Social media was identified as a platform where misinformation about menopause was a particular risk:

‘There is a lot of variability and false information especially in private Facebook groups’.

Another concern was the recent proliferation of information about menopause, which largely focused on negative aspects, resulting in anxiety:

‘A lot of information now scares women as they assume they are going to be faced with all symptoms whereas in practice all women experience the menopause and perimenopause differently’.

**Figure 1.**
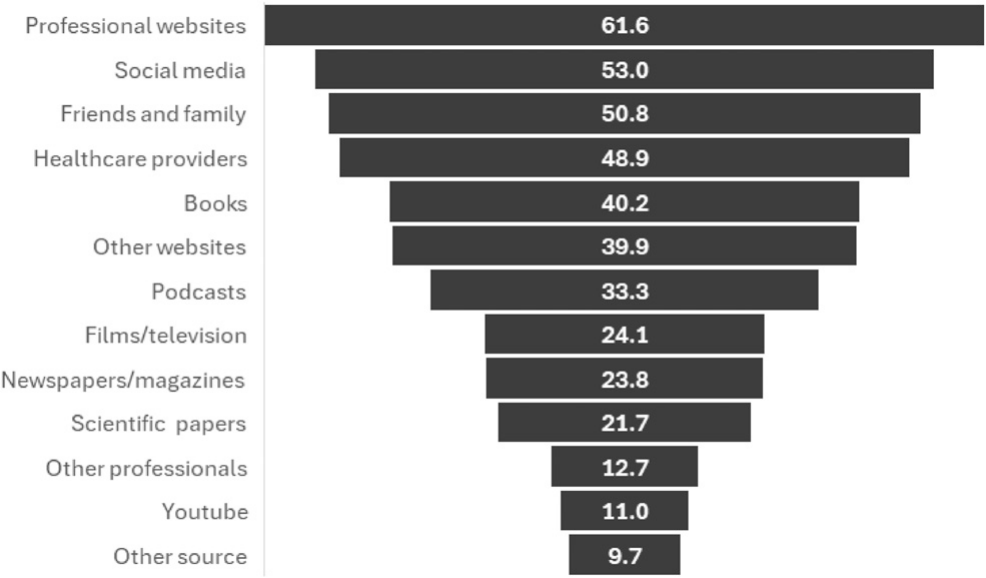
Different sources of information about menopause (proportion of respondents who reported accessing, *n* = 1596).

The vast majority of respondents (*n* = 1479, 92.7%) had sought information about menopause from a wide variety of sources (Figure 1). The most commonly accessed source of information was professional medical organisations (*n* = 983, 61.6%), followed by social media (*n* = 846, 53.0%, with Instagram the most popular) and friends and family (*n* = 811, 50.8%):

‘Everything I know about menopause I learnt on Instagram from other women’.

‘TikTok! The community spirit and support and sharing of information far outweighs any advice, proactive or otherwise, received from my Dr or OBGYN’.

Nearly half of participants had gone to a healthcare professional for information (*n* = 780). Many described feeling dismissed by healthcare professionals and/or receiving poor advice:

‘I’ve experienced a lot of doubt from the medical profession that my experience of being peri menopausal isn’t actual. There seems to be little information and no real care or time to offer options to the patient to choose their preferred treatment’.

‘I feel fairly confident I have begun to experience symptoms over the last 2–3 years, but every time I attempt to discuss this with a doctor I am dismissed with little explanation beyond age. It is frustrating and unclear’.

In contrast, support groups (often online) were an important source of information and help:

‘I only feel informed now because of the Facebook support groups I have joined’.

Those with long-term conditions, disability or who had experienced complex menopause (such as iatrogenic or early menopause) described a paucity of information tailored to their specific needs:

‘There is a lack of information for my circumstances - had early menopause due to surgery for ovarian cancer. Most women with ovarian cancer are older and postmenopause so the pathways are not in place for rarer cases’.

‘I’m autistic and postmenopausal. I feel invisible in both respects. The differences some autistic people experience going through menopause are not well understood, explained or provided for’.

For these populations, peer support was especially powerful, helping them understand the intersectional experiences of menopause in the context of other medical conditions:

‘I’m now part of a menopause diabetes group on SoMe [social media] which has helped me understand that I’m not the only one with certain symptoms and feelings’.

‘I joined menopause support groups and it really helped me understand what had happened because other people in the group had had hysterectomies. We were all angry that no-one gave us menopause info before the hysterectomy, so we knew the risks’.

Nearly two-thirds of respondents (*n* = 1024, 64.8%) reported that the information they accessed had been helpful, although many (*n* = 934, 59.1%) did not feel menopause support and information was tailored to their specific needs. Those from minoritised groups (sexually minoritised, gender diverse, ethnically minoritised and/or living with a disability or long-term condition) were less likely to find the information they had accessed useful (all *p* < .05). People living with a disability or long-term condition were more likely than those without a disability to report that current menopause resources were not tailored to them (*p <* .001), as were those who were ethnically minoritised, although this was not statistically significant (*p* = .087).

### Views and preferences for a national menopause education and support programme

There was broad support among respondents for a UK-wide menopause and education support programme, with 91.9% reporting that such a programme was needed (*n* = 1459). A minority were unsure what the proposed programme could offer beyond what already exists:

‘What more am I getting on a 8 week course that a leaflet or Google couldn't tell us’.

Many felt that menopause education needed to reach a broad section of society to foster greater understanding and support for people during this phase of life:

‘Everyone needs to know what this is that us women of a certain age are going through. I find people have less patience with women in their 50s etc. Young people especially but not only them, some men laugh at us, or I have been told to stop blaming everything on the menopause. Shop assistants are the worse, when I am experiencing brain fog in the supermarket they stand around me and act as if I am stealing something’.

‘Simply do it. Women’s health is deeply underrepresented and poorly researched. If you raise awareness that might have positive implications’.

### Programme delivery

The most popular mode of delivery for the proposed programme was either online and/or face-to-face in a group setting, with 92.0% (*n* = 1468) and 73.1% (*n* = 1166), respectively, expressing support for these options; only 742 (46.5%) reported a desire for an individual in-person programme. Accessing such a programme at work was less popular with 46.1% (*n* = 735), 27.6% (*n* = 441) and 53.3% (*n* = 850) saying they would want to attend in-person sessions in a group and/or individually, or online, via employers, respectively. Some participants had concerns that their employers would engage with the programme ‘Getting employers on board - the menopause is trivialised in many situations’ and some expressed concerns about delivery in the workplace ‘some people might not feel comfortable joining a program with colleagues’. Other suggestions included healthcare providers (including primary care, and at the over 40s health check), community women’s groups, gyms, and schools.

For the programme to support perimenopause symptoms, over half of participants replied that sessions should be 1 h long (58.3%, *n* = 921) with a further 21.1% (*n* = 334) stating they should be between one and one and a half hours (Table 2). The most popular options for frequency and number of sessions were once a week (*n* = 590, 37.8%) for between two and four sessions (*n* = 731, 46.8%). People highlighted the importance of acknowledging the competing priorities many people have at this stage in life, and the need to tailor the programme so it was not ‘time intensive’:

‘Menopausal women (like me) are often very time poor - we have jobs and families (children and parents) who rely on us for support. Please provide support in a format that isn't time intensive’.

**Table 2.** Preferences for programme delivery.

	*N*^a^ (%)
Duration of sessions (*n* = 1581)	
1 h	921 (58.3)
1.5 h	334 (21.1)
2 h	132 (8.4)
2.5 h	19 (1.2)
Other	175 (11.1)
Number of sessions (*n* = 1562)	
1	47 (3.0)
2–4	731 (46.8)
5–8	603 (38.6)
9–10	97 (6.2)
>10	84 (5.4)
Frequency of sessions (*n* = 1562)	
Once a week	590 (37.8)
Once every 2 weeks	507 (32.5)
Once a month	341 (21.8)
Other	124 (7.9)

^a^Numbers vary due to non-response.

Respondents identified engagement as a particular challenge, requiring careful thought about how to maintain interest over a number of weeks. One suggestion was to incorporate other activities, such as yoga or massage, so that sessions were fun:

‘If you incorporate some really fun or nurturing aspects to the training…maybe a yoga session at the end or an exercise session or a head massage or something, it could make it more appealing and show how important self-care is’.

Over two-thirds of respondents (*n* = 1091, 68.4%) said they would like the programme to be delivered by a non-healthcare provider (HCP) with menopause training, whereas 56.7% (*n* = 905) and 47.3% (*n* = 755) thought that a nurse or doctor should deliver it, respectively. Regardless of who delivered the programme, many respondents emphasised the need for facilitators to have appropriate training about menopause to avoid unintended harm:

‘An inadequate trainer will make people feel worse about themselves and the menopause. [You need to] ensure the trainer has a full and valid connection to the menopause’.

A few also indicated a preference for facilitators with lived experience of menopause:

‘I want to learn from people who have lived experience. I don’t want to be lectured by a 35-year-old doctor with no menopause experience, as my GP is’.

### Programme content

Respondents highlighted the challenge of developing a programme which could cater to the diversity of experiences of menopause and management strategies, which varies between individuals:

‘It seems everyone has their own symptoms [and] experiences so it might be hard to tailor everything but start with the basics and offer something separate on more specific topics’.

Others mentioned the importance of a holistic approach to menopause health and wellbeing, which does not focus on hormone replacement therapy (HRT) as the only solution:

‘The differences in women's experiences across the UK is huge. HRT isn’t for everyone and often works for a few months and then stops working…health issues aren't being picked up because HRT is thrown at everything’.

Another concern was the lack of awareness of menopause and its symptoms, which may result in individuals not identifying themselves as potentially eligible for a menopause education and support programme:

‘You have to know you are in menopause to do it [the programme]. A big problem is that people (particularly if they are younger) don’t know they are going through menopause. I therefore think there needs to be a lot of awareness made to younger people (in their 30 s) so that they are informed enough to identify when they may have entered perimenopause’.

**Figure 2.**
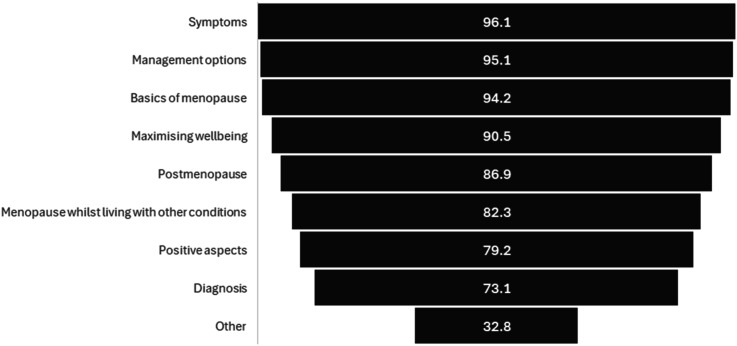
Topics to be covered in the programme (proportion of respondents who agreed, *n* = 1596).

We asked participants to indicate which topics they would like to see covered within the proposed programme; the most popular topics were menopause symptoms (*n* = 1533, 96.1%), management (*n* = 1517, 95.1%) and menopause physiological and social changes (*n* = 1503, 94.2%) (Figure 2). Other topics suggested included navigating menopause in the workplace and lesser-known symptoms.

Respondents stated a key focus must be ensuring information is accessible and accurate, and that the programme is based on ‘*latest scientific evidence and [aims to] debunk some of the myths*’. This was seen as especially important in the context of a perceived overwhelming amount of often conflicting information about menopause in the public domain.

Some participants expressed concern about potential conflicts of interest, and a need for a funding structure that guaranteed that advice was truly independent:

‘[There is a] risk of some companies funding and then skewing education to their advantage’.

### Building an inclusive programme

Participants emphasised the importance of developing a programme that was accessible to all, regardless of gender, ethnicity, age, sexuality, disability status and/or neurodivergence. They cited examples of groups who are currently neglected in discussions and support around menopause (including people who are socioeconomically marginalised, people from LGBTQIA+ communities, and those who living with neurodivergence). Experiences of exclusion among people from racially minoritised communities was particularly highlighted:

‘I feel in the UK that talks and studies tend to be about/by white people. Black people and POC [people of colour] aren’t as well represented, and our experiences of menopause can differ to [people of] white or other ethnicities’.

‘Women from ethnic minority backgrounds often feel their experiences are overlooked in menopause discussions’.

Participants believed that the programme ‘*needs to be accessible to all women regards of wealth, education or social position*’. Developing an inclusive programme that doesn’t amplify existing menopause health inequities, requires close partnership with affected communities:

‘How [do you] engage with those usually left out of the menopause conversation? Basically how to make it NOT “Mumsnet for Menopause.” This needs the invitation to also come from those within these communities - Black women, Asian women, queer people, disabled people, etc’.

One way of doing this is through co-design/co-production, a collaborative process where stakeholders, including end users and professionals, work together to develop an intervention, ensuring diverse perspectives are taken into account:

‘Definitely co-produce this course with people from different ethnicities, faith backgrounds, and ages who have lived experience of the condition’.

Respondents recommended that facilitators be from affected communities (i.e. living with a disability if delivering a programme to others living with a disability), or at the very least undergo training in inclusive practice such as cultural competence and gender inclusivity:

‘I think the trainers need to be trained on providing culturally competent information - women from different ethnicities may have different barriers or stigma around participating and sharing in a group or community forum’.

‘Make sure the trainers are properly trained, not only in the biological facts but in their attitudes. If they are not trained, for example, in gender variance/expansiveness, there is a risk of harm to trans and non-binary participants’.

Other suggestions to ensure the programme reached and met the needs of diverse groups included multilingual resources, multiple formats, step-free access, and British Sign Language (BSL) interpreters.

Some respondents identified neurodiversity as an often-overlooked factor that particularly impacted experiences of menopause and access to support:

‘Do keep in mind that most neurodivergent people in the main age range for perimenopause/menopause won’t have been diagnosed. Raising awareness of the possibility of this is really important as existing difficulties associated with being autistic or [having] ADHD [attention deficit hyperactivity disorder] can be exacerbated by hormonal fluctuations and social aspects of menopause’.

Material and delivery must therefore be designed to meet the needs of neurodiverse people, for example, considering online delivery.

Finally, respondents cautioned us to consider other groups who are often not included in current conversations and support around menopause. This included people who experience menopause at a young age, those who have experienced cancer, iatrogenic menopause, and those who have not had children:

‘Please acknowledge the added aspect of those childless women experiencing menopause - childless by choice or circumstance. [It] can be difficult for those who have never conceived to finally face the fact that this would no longer be biologically possible’.

## Discussion

Our survey found overwhelming appetite and support for a UK-wide menopause education and support programme. Most participants did not feel sufficiently informed or prepared for menopause, and only one-in-twenty expressed positive feelings about this stage of life. The lack of preparedness for and negative views towards menopause are consistent with findings from previous studies.^6–9,19,20^ The vast majority had sought information from a variety of sources including professional organisations and social media (especially Instagram). Respondents described navigating a complex and confusing landscape of conflicting information, which was often not tailored to those with complex medical needs and/or from marginalised groups.

Over 90% of respondents agreed that our proposed programme was needed, suggesting that it would also need to target younger individuals before they reach menopause and male partners, relatives and/or colleagues, as well as those experiencing menopause-related symptoms. This is similar to what we heard from our focus groups and workshops and is why we feel we need to offer two interventions; Be Prepared for Menopause and an intervention to support those going through perimenopause symptoms. Both programmes will cover what menopause is, symptoms, management, maximising wellbeing, living with other conditions, positive aspects, post menopause and diagnosis (Figure 2). There was broad consensus that the perimenopause symptoms programme could be delivered both in-person and online, in weekly hour-long sessions, over 2–4 weeks. Participants preferred facilitators to be non-healthcare professionals, but were clear that they should have menopause training and lived experience of menopause, as well as training in provision of inclusive support. The most popular topics to cover within the programme were menopause symptoms, management and physiological and social changes. Respondents emphasised the importance of all content being accurate, evidence-based and independent.

Psychosocial interventions including a combination of education, health promotion, decision-making support and/or cognitive behavioural therapy have been shown to reduce decisional conflict, and reduce menopausal and mood-related symptoms.^4^ A recent systematic review and meta-analysis has found a positive impact of menopause education on both quality of life and menopause symptomatology.^21^ However, it is important to note that only one of the eight studies included were conducted in Europe, and that most studies lacked ethnic diversity. Furthermore, there is a lack of longitudinal data, with follow-up data collected between one and 4 months post-intervention. There has been a rapid proliferation of menopause information and support in the UK and other settings. To the best of our knowledge, these programmes have not been co-designed with diverse communities and lack robust data on either efficacy or cost-effectiveness, which limits implementation on a national scale.

Peer support was highly valued by respondents, especially if they had long-term conditions and/or belonged to minoritised communities. Peer support has been shown to have positive outcomes in pregnancy and during breastfeeding,^22,23^ however, it is relatively under-studied in other areas of reproductive and post-reproductive health including the menopause transition. This highlights the need for robust evaluation of interventions that include peer support for menopause, to build an evidence base on this potentially cost-effective intervention which has minimal adverse effects.

Our findings underline the importance of recognising menopause as an intersectional experience. Respondents to our survey described widely varying lived experiences of menopause. Perceptions, symptoms, and care-seeking behaviours related to menopause vary by ethnicity,^24–28^ with evidence showing that individuals from racially minoritised communities face obstacles to accessing menopause care, including language barriers, limited health literacy, and systemic racism.^29,30^ Sexually minoritised and gender diverse individuals also experience discrimination and heteronormative assumptions within healthcare settings, and may encounter stigma and exclusion when accessing menopause support.^31–34^ Other populations who face barriers to menopause care (often a result of prior negative experiences in healthcare systems) are those who are neurodiverse and/or people living with mental health conditions.^35–38^

The dismissal of symptoms by healthcare providers, often disproportionately affecting the most marginalised individuals, reflects a broader issue of medical misogyny. This issue has been highlighted in a recent UK Parliamentary report^39^ and discussed extensively by physician Rageshri Dhairyawan in her book Unheard.^40^ It is therefore vital that any national programme of menopause education and support seeks to address the needs of *all* individuals experiencing menopause, in all their diversity. Respondents to our survey suggested that inclusivity could be fostered through practical steps such as co-design, partnership with grassroots organisations, ensuring venues and material are accessible, and training facilitators in inclusive practice.

Sampling bias is a key limitation to this study. Gender diverse, racially and sexually minoritised, and socioeconomically marginalised people are under-represented in our sample, and the questionnaire was only available in English. Consequently, we are unlikely to have captured fully their views and preferences. Sampling via personal and professional networks, and social media, mean that we may not have accessed marginalised groups, although the survey was circulated via grassroots organisations. Our qualitative data are limited to free-text responses, which do not allow for probing and are unlikely to capture the complexity of people’s views and experiences. Finally, as with all research conducted online, this study will have excluded people who experiencing digital inequities and who are not able to get online. The under-representation of marginalised communities in this study mean that our findings may not be applicable to these groups and may underestimate education and support needs.

It is important that our proposed intervention is guided by the lived experiences of people from these communities. This will ensure that the programme is acceptable and relevant, whilst avoiding reinforcing dominant cultural assumptions. We have conducted focus group discussions with diverse groups, including purposive sampling of people from racially minoritised communities, those with early menopause, gender diverse people, and people who are sexually minoritised (amongst others). These focus groups have allowed more detailed exploration of people’s needs and preferences regarding menopause education and support, and will inform the development of more inclusive, equitable and responsive menopause education and support (manuscript in preparation).

## Conclusion

After generations of silence, stigma and neglect, we are heartened to see menopause on policy and public health agendas. We have seen recent progress, for example, the inclusion of menopause education in the school curriculum.^41^ However, there is a clear gap for evidence-based and balanced menopause information and support at a national scale in the UK.^42^

Our proposed national programme of menopause education and support, InTune, seeks to fill this gap,^16^ combining free-to-access peer support and evidence-based information. Findings from this questionnaire study highlight significant unmet need for menopause information, especially among marginalised groups, and provide preliminary evidence of acceptability. Furthermore, we have elicited feedback on content and delivery of the programme which we will incorporate as we take this work forward.

With a doubling in NHS gynaecology waiting times and widespread pressures in primary care, there is an urgency to reconsider menopause education and support from a public health perspective. InTune is an innovative and ambitious programme that seeks to empower individuals to navigate this significant biological and social transition, enhancing health and wellbeing.

## Supplemental Material

Supplemental Material - ‘Simply do it.’: Results from an online questionnaire to inform a community-based menopause education and support programme in the UK, InTune Supplemental Material for “Simply do it.”: Results from an online questionnaire to inform a community-based menopause education and support programme in the UK, InTune by Joyce Catherine Harper, Nicky Keay, Mehab Mir, Annice Mukherjee, Jane Plumb, Geeta Kumar, Janet Lindsay, Jeremy Barratt, Sophie Strachan and Shema Tariq in Post Reproductive Health
