# Supplementary material for: ‘Simply do it.’: Results from an online questionnaire to inform a community-based menopause education and support programme in the UK, InTune
Source: Post Reprod Health. 2025 Sep 9;31(4):255–65. doi: 10.1177/20533691251372818 (PMC12669409; doi:10.1177/20533691251372818)
Supplement: Supplemental Material - ‘Simply do it.’: Results from an online questionnaire to inform a community-based menopause education and support programme in the UK, InTune [file sj-pdf-1-min-10.1177_20533691251372818.pdf]

Developing a UK menopause education and support programme

Start of Block: Consent

Q1
Developing a UK menopause education and support programme UCL Research Ethics Committee Approval: ID Number 9831/012 Principal Researcher: Professor Joyce Harper, 07880 795791, joyce.harper@ucl.ac.uk Department: Institute for Women's Health, Institute for Global Health, University College London Other team members: Shema Tariq, Nicky Keay, Polly Van Alstyne, Florrie Rowe Web site: https://www.ucl.ac.uk/global-health/uk-national-menopause-education-and-support-programme **We are inviting you to take part in an anonymous survey** • This survey is for anyone who has or could experience menopause (including women, transmen and non-binary people). • Please take time to read the following information carefully. Discuss it with friends and family, if you wish. Take time to decide whether or not you wish to take part. • You are free to decide whether or not to take part in this project. • You don't need to answer any question you don't want to answer. **1. Why are we doing this project?** Menopause is when periods stop and people can no longer become pregnant. It can be associated with a wide range of physical, mental health and social consequences. Approximately 13 million people in the UK are currently peri or postmenopausal; a 2019 survey found that nearly 1 million had left work because of their symptoms with major impact on individual lives and the economy. Our team has found that in a recent survey, over half of the women who responded were not aware of menopause and menopausal symptoms – this lack of information can mean that people have very negative feelings about menopause and don't know how best to manage through this life change. Women also say they don't currently have enough support during menopause and would like to meet other people at a similar life stage so they can share experiences. We want to help tackle this by designing the UK's first ever national menopause education and support programme but we need YOUR help. We want to make sure that anything we design is designed WITH people affected by menopause. Our aim is to develop a programme of support and education that is not for profit, evidence-based and inclusive, allowing people to access trusted information, share experiences and build a community. In this survey we'd like to ask you about where you get menopause information and support from, whether it has been enough, and what you think this new menopause programme should look like. This survey will complement focus groups we are doing with a diverse group of people affected by menopause. **2. Can I take part?** You need to be over 18 years old to take part. Anyone who has experienced or could potentially experience menopause can take part. It doesn't matter how old you are, or whether you have been through menopause yet. **3. What will I need to do if I take part?** If you decide to take part you will be asked to answer questions in an online survey. This will take about 15 minutes. You can withdraw at any time without giving a reason – simply do not submit your answers. If you decide to withdraw

before completing the survey, your answers will not be used in the study. Once you have submitted your answers, we cannot withdraw them as the survey is anonymous. **4. What are the possible disadvantages and risks of taking part?** It is very unlikely that you will come to any harm through completing this survey. This project has been approved by the UCL Research Ethics Committee. If any of the questions prompt you to want to know more about menopause you can access further information here: https://www.rcog.org.uk/for-the-public/menopause-and-later-life/, https://menopausesupport.co.uk/, www.menopausematters.co.uk/, https://www.themenopausecharity.org/. **5. What are the possible benefits of taking part in this study?** We cannot promise the study will help you personally, but you may enjoy knowing that you are helping us develop the UK's first national menopause education and support programme. This could help many people experiencing menopause in the future. You may also enjoy sharing your experiences and thoughts. **6. What if something goes wrong?** If you have any complaints about participating in this survey, you can contact to Professor Harper on joyce.harper@ucl.ac.uk or to the Chair of UCL Research Ethics Committee on ethics@ucl.ac.uk. In the unlikely event of something serious occurring during or following your participation in the project, please also contact Professor Harper. However should you feel your complaint has not been handled to your satisfaction please contact the Chair of UCL Research Ethics Committee. **7. What will happened to the information I provide?** Your answers will be anonymous. We will not be able to identify you. All the information that we collect about you during the course of the research will be kept strictly confidential. You will not be able to be identified in any reports or publications. Findings from the group discussion will help us develop the menopause education and support programme. We will use the opinions of people in these group discussions to guide the content and delivery of the programme. This will allow us to apply for more funding to be able to build the programme and test if it works. We will present some of the findings at academic conferences and publish results in academic journals, so that healthcare professionals and researchers can see them. The information will only be used for the purpose of improving menopause education and support and cannot be used to contact you. We will put the results on our study website: https://www.ucl.ac.uk/global-health/uk-national-menopause-education-and-support-programme The data from this survey will be stored for 10 years. We may share anonymous data with third parties on a case by case basis. 8 Local Data Protection Privacy Notice. The controller for this project will be University College London (UCL). The UCL Data Protection Officer provides oversight of UCL activities involving the processing of personal data, and can be contacted at data-protection@ucl.ac.uk This 'local' privacy notice sets out the information that applies to this particular study. Further information on how UCL uses participant information can be found in our 'general' privacy notice: For participants in health and care research studies, click here. The information that is required to be provided to participants under data protection legislation (GDPR and DPA 2018) is provided across both the 'local' and 'general' privacy notices. The lawful basis that will be used to process your personal data is: 'public task' and 'research purposes' will be the lawful basis for processing special category data. Your personal data will be processed so long as it is required for the research project. We will anonymise the personal data you provide. If you are concerned about how your personal data is being processed, or if you would like to contact us about your rights, please contact UCL in the first instance at data-protection@ucl.ac.uk
9. Who is organising and funding this study? The study has been designed by researchers

at University College London. The project is funded by an Innovation Network grant from UCL Innovation and Enterprise with funding from UK Research and Innovation (UKRI)'s Economic and Social Research Council (ESRC). UCL is Sponsor and has overall responsibility for the conduct of the group discussion part. Thank you for reading this information and for considering taking part. Your consent is important to us. Please can you click on the consent button below if you are happy to take part in this survey and please press submit if you want your answers included.

Q2 **Please read carefully:** **I am eligible because I am over 18 and I have experienced or could potentially experience menopause.** I have read the above information and understand what the study involves. I understand that if I decide at any time that I no longer wish to take part in this project, I can withdraw immediately by not submitting my answers. Once I have submitted my answers I will be unable to withdraw them. I understand that the information will be treated as strictly confidential and handled in accordance with the provisions of the General Data Protection Regulation, UK 2018. I understand that my fully anonymised data may be shared with other researchers. I have received all the information that I require, have indicated yes to all the above statements and consent to take part in this study. **Consent: I confirm that I am 18 or over. I have read and agree with the statements above**

○ Consent (1)

○ I do not consent (2)

Q3 There are three parts to this survey. You do not need to answer every question. We would like to ask you about yourself, your experience of menopause information and support and your views on what our menopause programme should be like. If you want to withdraw at any time, feel free to close the survey. We will not include your answers. We will only include your answers if you press submit. You will know when your survey results are submitted as you will be redirected to our web site. Thank you. **Part 1: Information about you We would like some basic information about you. This will help us see if we have got opinions from a diverse range of people.**

End of Block: Consent

Start of Block: Default Question Block

Page Break

Q4 **Please state your country of residence**

- ◯ UK (4)
- ◯ Other - please tell us more (5) ____

JS

Q5 **Please state your age in years:**

____

Q6 **Which describes you?**

- ◯ Female (1)
- ◯ Transman (4)
- ◯ Non-binary (5)
- ◯ Other (6)

Q8 **How would you describe your sexual orientation?**

- ◯ Heterosexual (1)
- ◯ Lesbian/gay (2)
- ◯ Bisexual (3)
- ◯ Pansexual (4)
- ◯ Asexual (5)
- ◯ Prefer not to say (6)

Q9 **Which of the following stages do you think best describes you?**

- ○ Premenopausal (my periods haven't changed and/or I haven't had any menopausal symptoms like hot flushes, vaginal dryness, mood changes, disturbed sleep) (1)
- ○ Perimenopausal (my periods have changed and/or I am getting some menopausal symptoms) (2)
- ○ Postmenopausal (I've not had a period for over a year) (3)
- ○ I am currently still having periods and I am not sure which stage I am in (5)
- ○ I do not have periods and I am not sure which stage I am in (6)
- ○ Not sure (7)

Q10 **Part 2: Menopause preparedness We would like to ask you some questions about your menopause experience and thoughts so we can work out the best way to design the UK menopause education and support programme.**

Q11 **How do you feel about the peri-menopause/menopause?**

- ○ Looking/looked forward to it (6)
- ○ Neutral - no strong view either way (8)
- ○ Feeling/felt negatively about it (9)
- ○ Not sure (10)
- ○ Have not thought about it (11)

Q12 **How informed do you feel about perimenopause/menopause?**

- ○ Very informed (1)
- ○ Somewhat informed (9)
- ○ Not informed at all (2)
- ○ Not sure (11)

Q13 **If you feel informed about menopause, what has helped you feel that way?**

____

____

____

____

____

Q14 **In the UK there are some public discussions about menopause. Do you feel these conversations reflect your experiences?**

- ○ Yes (1)
- ○ No (2)
- ○ Not sure (7)

Q15 **Do you feel that menopause information and support is tailored to you?**

- ○ Yes (1)
- ○ No - feel free to tell us more (2) ____
- ○ Not sure (3)

Q16 **Where have you accessed information about menopause in the past (tick all that apply).**

- ☐ I have not looked for information (10)
- ☐ Professional medical organisation web sites such as the NHS, British Menopause Society (14)
- ☐ Other web sites - feel free to tell us which web sites (1) ________________________________
- ☐ YouTube (2)
- ☐ Podcasts (13)
- ☐ Social media - feel free to tell us more about which social media (11) ________________________________
- ☐ Magazines (3)
- ☐ Newspapers (4)
- ☐ Books (5)
- ☐ Scientific papers (6)
- ☐ Films and TV programs (7)
- ☐ Friends and family (8)
- ☐ Health professionals (9)
- ☐ Other professional e.g therapist, trainer - feel free to tell us more about other professionals (16) ________________________________
- ☐ Other - feel free to tell us more about other sources (18) ________________________________

Q17 **Have you found the information you have accessed useful on the whole?**

- ◯ Yes (2)
- ◯ No (1)
- ◯ Not sure (4)
- ◯ Please tell us more (6) ____

Q18
We would like to know your opinions about the development of a UK menopause education and support programme. We aim to co-design this with the public, and this is why we are doing this survey. We plan to offer a range of packages including one-off sessions for people who haven't reached menopause yet, sessions for partners and a main programme which may be delivered over several weeks. There are many options, and we want to hear what you think.

Q19 **Does the UK need a national UK menopause and education support programme?**

- ▢ Yes (1)
- ▢ No (2)
- ▢ Not sure (3)
- ▢ Tell us more (4) ____

Q20 **Which topics should such a programme cover (please tick all that apply).**

- ☐ Basics of menopause: definitions, what and when to expect, physical, hormonal, emotional and social changes (1)
- ☐ Symptoms: for example changes in periods, hot flushes, urine infections, vaginal dryness, psychological issues (mood changes, brain fog, etc) (2)
- ☐ Menopause diagnosis (3)
- ☐ Maximising wellbeing: nutrition, exercise, sleep, psychological health, sex and relationships, friendships and community (4)
- ☐ Management options: hormone replacement therapy, vaginal treatments, talking therapies, alternative therapies, testosterone, other medication (including newer treatments) (5)
- ☐ Menopause when you have other conditions: early menopause, surgical menopause, surviving cancer (6)
- ☐ Postmenopause: how to stay well beyond menopause (7)
- ☐ Positive aspects of menopause (8)
- ☐ Any other topics we have missed out? Please tell us more... (9) ____

Q21 **How do you think we could deliver menopause education and support (please tick all that apply)?**

- ☐ Online (1)
- ☐ In person, in a group (2)
- ☐ In person, one-to-one (7)
- ☐ Through your employer online (3)
- ☐ Through your employer, in person, in a group (4)
- ☐ Through your employer, in person, one-to-one (8)
- ☐ Other - please tell us more (5) ____

Q22 **The MAIN package will be aimed at people going through menopause. For the MAIN package, which may be about 8 sessions (still to be confirmed), how long should each of the sessions last?**

- ○ 1 hour (1)
- ○ 1.5 hours (2)
- ○ 2 hours (3)
- ○ 2.5 hours (4)
- ○ Other - please tell us more (5) ____

Q23 **For the MAIN package, how many sessions would be enough to cover the important issues and be practical for people to attend?**

○ 1 (1)

○ 2-4 (2)

○ 5-8 (3)

○ 9-10 (4)

○ More than 10 - how many? (5) ____

Q24 **For the MAIN package - how often should these sessions be?**

○ Once a week (1)

○ Once every two weeks (2)

○ Once a month (3)

○ Other - please tell us more (4) ____

Q25 **Who should deliver a UK menopause education and support programme (please tick all that apply)?**

- ☐ Doctor (1)
- ☐ Nurse (2)
- ☐ Other healthcare professional (3)
- ☐ Non healthcare professional with menopause expertise (with training) (4)
- ☐ Other (6)
- ☐ Please tell us more (5) ____

Q26 **What challenges could we face when setting up a suite of programmes like this? How could we tackle these? Free text answer.**

____

____

____

____

____

Q27 **Is there anything else you'd like to share about this proposed national UK menopause education and support programme, especially anything we've forgotten to ask about? Please don't include your contact details as this should be an anonymous survey.**

____

____

____

____

Q28 **Final section: About you (part II) Part 3: We have a few final questions to ask about your background. Remember all your answers are anonymous. We will only include your answers if you press submit at the end of this section. You will know when the survey is submitted as you will receive information about the menopause.**

Page Break

Q29 **What is your highest educational qualification?**

- ○ Did not attend school (5)
- ○ Primary school (8)
- ○ Secondary School - A levels/GCSEs, etc (1)
- ○ Sixth form/A Level/College-level (6)
- ○ University undergraduate (2)
- ○ University postgraduate (3)
- ○ Other (4) ____

Q30 **How do you identify yourself? Choose one or more.**

- ☐ White - English / Welsh / Scottish / Northern Irish / British (1)
- ☐ White - Irish (2)
- ☐ Any other White background (please specify) (3) ____
- ☐ Black/Black British - African (4)
- ☐ Black/Black British - Caribbean (10)
- ☐ Any other Black/African/Caribbean background (please specify) (11) ____
- ☐ Latino (16)
- ☐ Asian/Asian British - Indian (12)
- ☐ Asian/Asian British - Pakistani (13)
- ☐ Any other Asian background (please specify) (5) ____
- ☐ Arab (8)
- ☐ Mixed ethnic background (please specify) (7) ____
- ☐ Any other ethnic group, please describe (9) ____
- ☐ Prefer not to say (15)

Q31 **I have enough money to cover my basic needs (e.g. food, housing, heating).**

- All of the time (1)
- Most of the time (2)
- Some of the time (3)
- None of the time (4)

Q32 **What is your disability status?** The Equality Act 2010 states a person has a disability if they have a physical or mental impairment that has a substantial and long-term adverse effect (likely to last 12 months or more) on their ability to perform normal day-to-day activities (e.g. eating, washing, walking and going shopping).

- No disability (1)
- Sensory impaired (2)
- Physical or mobility impaired (3)
- Specific learning difficulty or disability (e.g. dyslexia) (4)
- General learning disability (cognitive) (5)
- Long term illness or health condition (6)
- Autistic spectrum disorder (7)
- Other, please specify (8) ____
- Prefer not to say (9)

Page Break

Q33 Thank you for your time in completing our survey. We value and appreciate your participation. When you have read this paragraph, please press the next arrow and your answers will then be included in our study and you will be redirected to our web site. If you do not press the final arrow, your answers will not be included.   If you would like to discuss any of the issues that came up in this questionnaire, please know that there are sources of support available. In the first instance, you may wish to contact your doctor.   Other sources of information and support include: https://www.nhs.uk/conditions/menopause/ https://www.womens-health-concern.org/ https://www.themenopausecharity.org/ https://www.menopausematters.co.uk/ https://www.queermenopause.com/resources https://www.thekarenarthur.com/menopausewhilstblack https://www.daisynetwork.org/

End of Block: Default Question Block

Start of Block: Block 2
